# Supplementary material for: Recognition and control of neutrophil extracellular trap formation by MICL
Source: Nature. 2024 Aug 14;633(8029):442–50. doi: 10.1038/s41586-024-07820-3 (PMC11390483; doi:10.1038/s41586-024-07820-3)
Supplement: Supplementary file 1 — Reporting Summary [file 41586_2024_7820_MOESM1_ESM.pdf]

nature portfolio

Corresponding author(s): Gordon D. Brown

Last updated by author(s): Jun 14, 2024

Reporting Summary

Nature Portfolio wishes to improve the reproducibility of the work that we publish. This form provides structure for consistency and transparency in reporting. For further information on Nature Portfolio policies, see our Editorial Policies and the Editorial Policy Checklist.

Statistics

For all statistical analyses, confirm that the following items are present in the figure legend, table legend, main text, or Methods section.

n/a	Confirmed	
☐	☒	The exact sample size (n) for each experimental group/condition, given as a discrete number and unit of measurement
☐	☒	A statement on whether measurements were taken from distinct samples or whether the same sample was measured repeatedly
☐	☒	The statistical test(s) used AND whether they are one- or two-sided<br>*Only common tests should be described solely by name; describe more complex techniques in the Methods section.*
☐	☒	A description of all covariates tested
☐	☒	A description of any assumptions or corrections, such as tests of normality and adjustment for multiple comparisons
☐	☒	A full description of the statistical parameters including central tendency (e.g. means) or other basic estimates (e.g. regression coefficient) AND variation (e.g. standard deviation) or associated estimates of uncertainty (e.g. confidence intervals)
☒	☐	For null hypothesis testing, the test statistic (e.g. F, t, r) with confidence intervals, effect sizes, degrees of freedom and P value noted<br>*Give P values as exact values whenever suitable.*
☒	☐	For Bayesian analysis, information on the choice of priors and Markov chain Monte Carlo settings
☒	☐	For hierarchical and complex designs, identification of the appropriate level for tests and full reporting of outcomes
☐	☒	Estimates of effect sizes (e.g. Cohen's d, Pearson's r), indicating how they were calculated

Our web collection on statistics for biologists contains articles on many of the points above.

Software and code

Policy information about availability of computer code

Data collection	For Flow cytometry data collection Becton Dickinson FACSDiva v.8.0.3, SpectroFlo v.3.2.1 or INSPIRE software was used.
Data analysis	GraphPad Prism v.10.2.0 was used for statistical analysis<br>FlowJo v.10 was used for flow cytometry data analysis<br>Fiji was used for microscopy image analysis<br>QuPath v.0.4.3 was used for segmentation of fluorescent microscopy images

For manuscripts utilizing custom algorithms or software that are central to the research but not yet described in published literature, software must be made available to editors and reviewers. We strongly encourage code deposition in a community repository (e.g. GitHub). See the Nature Portfolio guidelines for submitting code & software for further information.

Data

Policy information about availability of data

All manuscripts must include a data availability statement. This statement should provide the following information, where applicable:

- Accession codes, unique identifiers, or web links for publicly available datasets
- A description of any restrictions on data availability
- For clinical datasets or third party data, please ensure that the statement adheres to our policy

All data necessary for the conclusions of this study are available in the main text, figures and Extended data. Source data are provided with the Article. Additional RA

patient data is available on request from the Scottish Early Rheumatoid Arthritis (SERA) and approval by the SERA Access Committee (Dale, J., Paterson, C., Tierney, A. et al. The Scottish Early Rheumatoid Arthritis (SERA) Study: an inception cohort and biobank. BMC Musculoskelet Disord 17, 461 (2016)).

Research involving human participants, their data, or biological material

Policy information about studies with human participants or human data. See also policy information about sex, gender (identity/presentation), and sexual orientation and race, ethnicity and racism.

Reporting on sex and gender	Based on biological attributes provided about the cohort from the Scottish Early Rheumatoid Arthritis (SERA) from the 200 samples used, 123 (61.5%) were females and 77 (38.5%) were males.
Reporting on race, ethnicity, or other socially relevant groupings	None of these characteristics were included in the data collected from participants of the SERA cohort (Dale, J., Paterson, C., Tierney, A. et al. The Scottish Early Rheumatoid Arthritis (SERA) Study: an inception cohort and biobank. BMC Musculoskelet Disord 17, 461 (2016))<br>None of these characteristics were included in the data collected from participants of the Covid-19 cohorts (Kusakabe T, Lin WY, Cheong JG, Singh G, Ravishankar A, Yeung ST, Mesko M, DeCelie MB, Carriche G, Zhao Z, Rand S, Doron I, Putzel GG, Worgall S, Cushing M, Westblade L, Inghirami G, Parkhurst CN, Guo CJ, Schotsaert M, García-Sastre A, Josefowicz SZ, Salvatore M, Iliev ID. Fungal microbiota sustains lasting immune activation of neutrophils and their progenitors in severe COVID-19. Nat Immunol. 2023 Nov;24(11):1879-1889. doi: 10.1038/s41590-023-01637-4)
Population characteristics	From the 200 samples from the Scottish Early Rheumatoid Arthritis (SERA) Study used, patients were 58 ±13 years old, 123 (61.5%) were females and 77 (38.5%) were males.<br>For the Covid-19 cohort, participants were recruited from the inpatient division of New York-Presbyterian Hospital: 25 moderate COVID-19 patients (10 males and 15 females, median age of 61.7 years), 66 severe COVID patients-19 (45 males and 21 females, median age of 66.6 years) and 36 healthy controls (16 males and 20 females, median age of 54.5 years).<br>For the SLE cohort, samples were obtained from the Imperial College Healthcare Tissue Bank (ICHTB). 40 SLE patients (40 females, median age 42.5 years) and 27 healthy controls (27 females, median age 36 years)
Recruitment	"Rheumatology units from across Scotland participate in the SERA study. Patients with a new clinical diagnosis of RA or UA, and who have at least one swollen joint, are invited to participate. Patients are excluded if their joint swelling can be explained by an alternative diagnosis (e.g. psoriatic arthritis) or if they are carriers of blood borne viruses. Duration of symptoms up until diagnosis is not an exclusion criterion. Potential participants are referred to local SERA research nurses for screening and baseline assessments. Treatment decisions (including initiation and escalation) and clinical follow-up remain the responsibility of the local rheumatology department who follow standard local practice. Patients are not excluded if treatment with steroids or DMARDs has already started prior to recruitment (for example by the General Practitioner) as long as the diagnosis of UA/RA is new, and treatment has commenced within the last 6 months. Participants are asked to invite a first degree relative, or friend of the same gender and similar age, to participate in a cohort of healthy controls with a similar genetic or demographic background. All participants provide generic and enduring consent that allows: collection of demographic and outcome data; retrieval and linkage of routine health care data; and long term storage of data and samples for future research projects."(Dale, J., Paterson, C., Tierney, A. et al. The Scottish Early Rheumatoid Arthritis (SERA) Study: an inception cohort and biobank. BMC Musculoskelet Disord 17, 461 (2016))<br>"Recruitment of Covid-19 samples: Participants were recruited from patients hospitalized at New York Presbyterian Hospital from March to June 2020. Some subjects were followed after recovery from mCOVID-19 or sCOVID-19 and were partitioned into an early convalescent group (2-4 months following admission) and a late convalescent group (4-12 months following admission). All patients were classified in mCOVID-19 and sCOVID-19 according with oxygen requirements with mCOVID-19 defined as SARSCoV-2 infection and <6 liters noninvasive supplemental oxygen to maintain SpO2 >92%, and sCOVID-19 defined as SARS-CoV-2 infection requiring hospitalization and received >6 liters supplemental oxygen or mechanical ventilation (Kusakabe T, Lin WY, Cheong JG, Singh G, Ravishankar A, Yeung ST, Mesko M, DeCelie MB, Carriche G, Zhao Z, Rand S, Doron I, Putzel GG, Worgall S, Cushing M, Westblade L, Inghirami G, Parkhurst CN, Guo CJ, Schotsaert M, García-Sastre A, Josefowicz SZ, Salvatore M, Iliev ID. Fungal microbiota sustains lasting immune activation of neutrophils and their progenitors in severe COVID-19. Nat Immunol. 2023 Nov;24(11):1879-1889. doi: 10.1038/s41590-023-01637-4)<br>All patients with SLE in the study met the revised American College of Rheumatology criteria [Tan EM, et al. The 1982 revised criteria for the classification of systemic lupus erythematosus. Arthritis Rheum. 25, 1271-1277 (1982)] and the SLICC [Petri M, et al. Derivation and validation of the Systemic Lupus International Collaborating Clinics classification criteria for systemic lupus erythematosus. Arthritis Rheum. 64, 2677-2686 (2012)] criteria. Some patient had a history of biopsy-proven nephritis according to the International Society of Nephrology/Renal Pathology Society classification. Healthy female volunteers (with no family history of autoimmune disease) served as age-matched and ethnicity-matched controls.
Ethics oversight	"The SERA Study was initiated by the Scottish Collaborative Arthritis Research (SCAR, www.scarnetwork.org) network and represents a collaboration between the Universities of Aberdeen, Dundee, Edinburgh and Glasgow, NHS Scotland, Healthcare Improvement Scotland, the Chief Scientist's Office Scotland and Pfizer Ltd. The study's protocol and procedures were reviewed and given favourable opinion by the West of Scotland Research Ethics Committee and all included patients provided written, enduring consent to participate. The study is managed by a scientific steering committee comprising clinicians and academics, from each of the participating NHS Health Boards and Universities, and (until April 2015) representatives of Pfizer Ltd." (Dale, J., Paterson, C., Tierney, A. et al. The Scottish Early Rheumatoid Arthritis (SERA) Study: an inception cohort and biobank. BMC Musculoskelet Disord 17, 461 (2016))<br>-Serum samples were also obtained from consenting healthy donors with the approval of the College of Life Sciences and Medicine ethics review board, University of Aberdeen (Application number 1243)<br>"Covid-19 patients research was reviewed and approved by the Institutional Review Board of Weill-Cornell Medicine (New York Presbyterian and Lower Manhattan hospitals) (#IRB 20-03021645 and #IRB 20-03021671). Informed consents were obtained from all enrolled patients and healthcare workers by trained staff and records maintained in research database for the duration of our study" (Kusakabe T, Lin WY, Cheong JG, Singh G, Ravishankar A, Yeung ST, Mesko M, DeCelie MB, Carriche

G, Zhao Z, Rand S, Doron I, Putzel GG, Worgall S, Cushing M, Westblade L, Inghirami G, Parkhurst CN, Guo CJ, Schotsaert M, García-Sastre A, Josefowicz SZ, Salvatore M, Iliev ID. Fungal microbiota sustains lasting immune activation of neutrophils and their progenitors in severe COVID-19. Nat Immunol. 2023 Nov;24(11):1879-1889. doi: 10.1038/s41590-023-01637-4)
For the SLE cohort, all patients gave informed consent and samples used in this research project were obtained from the Imperial College Healthcare Tissue Bank (ICHTB). ICHTB is supported by the National Institute for Health Research (NIHR) Biomedical Research Centre based at Imperial College Healthcare NHS Trust and Imperial College London. ICHTB is approved by Wales REC3 to release human material for research (22/WA/0214), and the samples for this project (Ref: R13010a) were issued from sub-collection reference number IMM_MB_13_001.

Note that full information on the approval of the study protocol must also be provided in the manuscript.

Field-specific reporting

Please select the one below that is the best fit for your research. If you are not sure, read the appropriate sections before making your selection.

☒ Life sciences ☐ Behavioural & social sciences ☐ Ecological, evolutionary & environmental sciences

For a reference copy of the document with all sections, see nature.com/documents/nr-reporting-summary-flat.pdf

Life sciences study design

All studies must disclose on these points even when the disclosure is negative.

Sample size	Sample sizes of at least five per group were chosen as this would allow the detection of a 25% difference in the mean between experimental and control groups with a probability of greater than 95% (p < 0.05), assuming a standard deviation of around 15% and a minimum power value of 0.8.
Data exclusions	Extended Data Fig 1a. One mouse was excluded from analysis due to lack of response<br>Extended data Fig 6c. 3 animals were excluded since they were euthanized during the experiment
Replication	All experiments were independently replicated at least once unless otherwise indicated in the manuscript.
Randomization	Mouse experiments were performed by random assignation of age- and sex-matched mice in experimental groups at the beginning of each experiment to experimental or control groups, females were co-housed, and experiments were not blinded.<br>For in vitro experiments, samples were randomly assigned to experimental or control groups.
Blinding	Experiments were not blinded, as the investigator who planned the experiments, also performed them but were conducted according to standardized protocols and procedures.

Reporting for specific materials, systems and methods

We require information from authors about some types of materials, experimental systems and methods used in many studies. Here, indicate whether each material, system or method listed is relevant to your study. If you are not sure if a list item applies to your research, read the appropriate section before selecting a response.

Materials & experimental systems

n/a	Involved in the study	
☐	☒	Antibodies
☐	☒	Eukaryotic cell lines
☒	☐	Palaeontology and archaeology
☐	☒	Animals and other organisms
☒	☐	Clinical data
☒	☐	Dual use research of concern
☒	☐	Plants

Methods

n/a	Involved in the study	
☒	☐	ChIP-seq
☐	☒	Flow cytometry
☒	☐	MRI-based neuroimaging

Antibodies

Antibodies used	anti-CD45-FITC (Clone 102), anti-CD45-PerCP-Cyanine5.5(Clone 102), anti-CD11b-BUV395(Clone M1/70), anti-CD11b-PE-Cy7 (Clone M1/70), anti-GR-1-APC (Clone RB6-8C5), anti-MHC-II-FITC (Clone 2G9), anti-MHC-II-BUV496 (Clone 2G9)anti-C5aR-BV510 (Clone 20/70), anti-Ly6G-BV421(Clone 1A8), anti-Ly6G-Spark Blue 550(Clone 1A8), anti-Ly6GAPC (Clone 1A8), anti-CD62L-BV510 (Clone MEL-14), anti-CD18-BV650 (Clone C71/16), anti-CD18-APC (H155-78), anti-F4/80-AF700 (Clone BM8), anti-F4/80-PECy7 (Clone BM8), anti-Ly6C-PE-Cy7 (Clone HK1.4), anti-Ly6C-Brilliant Violet 570 (Clone HK1.4), anti-CCR1-PE (Clone 643854), anti-CXCR2-APC (Clone SA045E1), anti-CD11c-BV711 (Clone HL3), anti-B220-AlexaFluor700 (Clone RA3-6B2), isotype control AFRC MAC 49 (ECACC 85060404; isotype for anti-MICL), anti-CD66-PE/Dazzle (Clone G10F5), anti-CD15-AF700 (Clone Hi98), anti-CD16-APC (Clone 3G8) and HRP-conjugated goat F(ab')2 fragment anti-human IgG (JacksonImmuno Research). Remaining antibodies were purchased commercially from eBioscience, R&D systems or Biolegend.

anti-mMICL (309), isotype control AFRC MAC 49 (ECACC 85060404; isotype for anti-mMICL), anti-hMICL (HB3) and isotype control D1.3 (isotype for anti-hMICL) were generated in house.
anti-citrullinated histone 3 (ab5103, Abcam), anti-Myeloperoxidase (AF3667, R&D) and anti-DNA/H1 (AB3864, Merck) were used for Immunofluorescence staining of NETs
To achieve neutrophil depletion rat anti-mouse Ly6G (Clone 1A8), rat anti-mouse GR-1 (Clone RB6-8C5) or isotype controls (rat anti-mouse IgG2a, rat anti-mouse IgG2b). All were purchased commercially from Bio-X-cell.

Validation

Anti-mMICL antibodies were generated and validated as follows. Sprague Dawley rats were immunised with Fc-mMICL in Freund's complete adjuvant. After a final intraperitoneal boost, without adjuvant, rat splenocytes were harvested and fused with Y3 myeloma cells, as described. Hybridoma supernatants were screened by ELISA and positives were then tested by immunohistochemistry and flow cytometry, as described below, against Fc-mMICL as well as mMICL transduced NIH3T3 fibroblasts.
The mAb, HB3, specific for hMICL, was generated by immunization of C57BL/6 mice with an Fc-hMICL fusion protein. Hybridomas were generated according to standard protocols and supernatants from clonally diluted cells were screened by ELISA. The mAb HB3 (IgG1) was subsequently selected based on its ability to function in FACS, Western blot, and immunocytochemistry.
Remaining antibodies were well validated commercial clones and routinely QC'ed by the manufacturer.Please refer to the spec sheets on the respective vendors' website for technical information and detail by searching the catalog numbers or clone numbers provided above.

Eukaryotic cell lines

Policy information about cell lines and Sex and Gender in Research

Cell line source(s)

HEK293T, NIH3T3 cell lines were originally purchased from the ATCC. BWZ.36 NFAT-lacZ were kindly provided by Wayne Yokoyama, Washington, USA

Authentication

No authentication methods were used.

Mycoplasma contamination

Mycoplasma tests were done historically on these cell lines, but not for the experiments detailed in the manuscript.

Commonly misidentified lines
(See ICLAC register)

none are listed as misidentified

Animals and other research organisms

Policy information about studies involving animals; ARRIVE guidelines recommended for reporting animal research, and Sex and Gender in Research

Laboratory animals

C57BL/6 and Clec12a -/- mice (6-8 weeks old) were obtained from the specific pathogen-free facility at the University of Aberdeen or Charles River Laboratories. Animal experiments were performed using age-matched female or male mice and conformed to the animal care and welfare protocols approved by UK Home Office (Project license numbers: P79B6F297 and P6A6F95B5) in compliance with all relevant local ethical regulations. Clec12a-/- mice were generated commercially (Taconic Artemis) by conventional gene targeting in C57BL/6 embryonic stem cells (Redelinghuys P, Whitehead L, Augello A, et al. MICL controls inflammation in rheumatoid arthritis, Annals of the Rheumatic Diseases 2016;75:1386-1391).
Male DBA/1OlaHsd mice (6-8) weeks old were obtained from Inotiv and maintained at the University of Exeter.

Wild animals

The study did not involve wild animals

Reporting on sex

Collagen type II antibody-induced arthritis (CAIA) and Collagen-induced arthritis (CIA) were originally described in males as they have have enhanced susceptibility compared to females (Holmdahl R, Jansson L, Larsson E, Rubin K, Klareskog L: Homologous type II collagen induces chronic and progressive arthritis in mice. Arthritis Rheum 1986, 29:106-113; Nandakumar KS, Svensson L, Holmdahl R. Collagen type II-specific monoclonal antibody-induced arthritis in mice: description of the disease and the influence of age, sex, and genes. Am J Pathol. 2003).
Data in this paper shows CAIA, CIA and K/BxN serum transfer models performed in males. CAIA data was also confirmed in females.

Field-collected samples

The study did not involve samples collected from the field

Ethics oversight

All experiments conformed to the ethical review committee of the University of Aberdeen, University of Exeter, and the UK Home Office regulations (Project license numbers: P79B6F297 and P6A6F95B5).

Note that full information on the approval of the study protocol must also be provided in the manuscript.

Flow Cytometry

Plots

Confirm that:

☒ The axis labels state the marker and fluorochrome used (e.g. CD4-FITC).

☒ The axis scales are clearly visible. Include numbers along axes only for bottom left plot of group (a 'group' is an analysis of identical markers).

☒ All plots are contour plots with outliers or pseudocolor plots.

☒ A numerical value for number of cells or percentage (with statistics) is provided.

Methodology

Sample preparation

Murine cells and tissues:
Murine peripheral blood leukocytes, thioglycolate-elicited inflammatory peritoneal cells and bone marrow cells were isolated essentially as described previously (Taylor PR et al Eur J Immunol, 2003).
Bone marrow neutrophils were isolated using a gradient of Histopaque separation media (Merck) by a density gradient centrifugation method or using the EasySep™ Mouse Neutrophil Enrichment Kit (STEMCELL Technologies).
Cells were isolated from the hind paw ankle joint of arthritic mice using the protocol described by Armaka et al 2009. The isolated tissue was incubated 60 minutes with Collagenase VIII (Sigma-Aldrich). Cells were strained through 70 µm nylon cell strainers (Fisher Scientific) and collected by centrifugation.

NNIH3T3 fibroblasts stably expressing full length murine or human MICL were maintained at 37°C and 5% CO2 in DMEM or RPMI medium supplemented with 10% heat-inactivated foetal calf serum, 100 units/mL penicillin, 0.1 mg/mL streptomycin, and 2 mM L-glutamine.

For flow cytometry, single cell suspensions were stained with fixable viability dye efluor 780 (eBioscience), and washed in FACS wash (PBS with 0.5% (w/v) BSA and 5-10 mM EDTA) containing anti-CD16/CD32 (Clone 2.4G2, prepared in house). The following antibodies were used for FACS analysis following standard methodology: anti-CD45-FITC (Clone 102), anti-CD45-PerCP-Cyanine5.5(Clone 102), anti-CD11b-BUV395(Clone M1/70), anti-CD11b-PE-Cy7 (Clone M1/70), anti-GR-1-APC (Clone RB6-8C5), anti-MHC-II-FITC (Clone 2G9), anti-MHC-II-BUV496 (Clone 2G9), anti-C5aR-BV510 (Clone 20/70), anti-Ly6G-BV421(Clone 1A8), anti-Ly6G-Spark Blue 550(Clone 1A8), anti-Ly6GAPC (Clone 1A8), anti-CD62L-BV510 (Clone MEL-14), anti-CD18-BV650 (Clone C71/16), anti-CD18-APC (H155-78), anti-F4/80-AF700 (Clone BM8), anti-F4/80-PECy7 (Clone BM8), anti-Ly6C-PE-Cy7 (Clone HK1.4), anti-Ly6C-Brilliant Violet 570 (Clone HK1.4), anti-CCR1-PE (Clone 643854), anti-CXCR2-APC (Clone SA045E1), anti-CD11c-BV711 (Clone HL3), anti-B220-AlexaFluor700 (Clone RA3-6B2), and anti-CD3-Alexa Fluor 647 (Clone 17A2) a. All were purchased commercially from eBioscience, R&D systems or Biolegend. αmClec12a-biotinylated, and isotype control AFRC MAC 49 (ECACC 85060404; isotype for anti-mMICL) was generated in house.

Human neutrophils from blood of healthy donors were purified using a Ficoll-Paque density centrifugation method (Kuhns DB et al, Curr. Protoc. Immunol. 2015) or using the EasySepTM direct human neutrophil isolation kit (STEMCELL Technologies) as per the manufacturer's instructions. Single cell suspensions were stained with fixable viability dye Zombie Aqua. Cells were washed in FACS wash (PBS with 0.5% (w/v) BSA and 5-10 mM EDTA) and stained with anti-CD66-PE/Dazzle (Clone G10F5), anti-CD15-AF700 (Clone Hi98), anti-CD16-APC (Clone 3G8) and anti-hMICL or isotype control antibodies. All were purchased commercially from Biolegend. αhMICL was generated in house.

Instrument

BD LSR II Fortessa flow cytometer (BD Biosciences), Cytek Aurora Spectral cytometer (Cytek), Amnis Image StreamX imaging flow cytometer

Software

For collection Becton Dickinson FACSDiva, SpectroFlo software and INSPIRE software were used. For analysis FlowJo software was used.

Cell population abundance

No cell populations were sorted for this manuscript.

Gating strategy

Gating strategy for murine tissue staining
Using the FSC/SSC plot, a gate was drawn to select cells and exclude debris.
Using the FSC-H/FSC-A plot, a gate was drawn to select single cells.
Using the FSC-H/Efluor780 plot, a gate was drawn to select viable cells.
Using the FSC-H/ PerCP-Cy5.5 plot, we gated on CD45 negative and positive cells.
Using the Spark Blue 550/BUV395 plot, we gated on Ly6G CD11b double positive cells, CD11b positive Ly6G negative cells and Ly6G negative CD11b negative cells.
From the D11b positive Ly6G negative cells, using the PECy7/ BUV395 we gated on F4/80 positive cells.
From the F4/80 positive cells, using the Brilliant Violet 570 /BUV395 we gated on Ly6C high and Ly6C low cells.
From the Ly6G negative CD11b negative cells, using the BV605/Alexa Fluor 647 we gated on B220 positive or CD3 positive cells.
Positive and negative populations for gating were defined using FMO and isotype controls.

Gating strategy for cultured neutrophils
Using the FSC/SSC plot, a gate was drawn to select cells and exclude debris.
Using the FSC-H/FSC-A plot, a gate was drawn to select single cells.
Using the FSC-H/BV510 plot, a gate was drawn to select viable cells.

Using the PE-Dazzle/Alexa Fluor 700 plot we gated on CD66b CD15 double positive cells. We used this gate to detect MICL expression using the Alexa Fuor 488 channel.

☒ Tick this box to confirm that a figure exemplifying the gating strategy is provided in the Supplementary Information.
